# Cumulative Dis/Advantage in a Changing World: Structural and Policy Impacts

**DOI:** 10.1093/geroni/igaf122.556

**Published:** 2025-12-31

**Authors:** Dale Dannefer, Stephen Crystal

## Abstract

.In a rapidly-changing world, improving our understanding of late-life inequality and the structural and policy factors operating over the lifecourse to drive these outcomes is vital. Research on cumulative advantage/disadvantage processes has provided important insights on these factors, but much remains to be understood. Additional insights are emerging from new research approaches. These include comparative analyses of inequality across age groups in countries with varying retirement income systems, and longitudinal analyses of the role of structural factors including marital histories, residential segregation, and education, interacting with policy factors such as retirement income systems, in shaping disparate life-course outcomes. This symposium will include present several such analyses of the operation of social processes underlying cumulative dis/advantage. Papers will cover several fresh directions of empirical research, including comparative analyses that present comparisons across age groups and cross-national comparisons (Crystal, Zhou), and evidence on mechanisms linking education (Kim, Zhou) and family (Carr) factors to inequality in mental and physical health outcomes. The symposium will also include a fresh critical analysis and assessment of the uses and misuses of cumulative dis/advantage in the gerontological and life-course literatures and trends in emerging scholarship (Kelley). Empirical contributions will address the role of midlife segregation and healthcare access as mediators of education and cognitive impairment; the role of marital histories interacting with the US Social Security system in contributing to older adults’ economic outcomes; and cross-national analyses of late-life inequality before, during, and after the COVID pandemic.

